# The Cohesive Energy and Vibration Characteristics of Parallel Single-Walled Carbon Nanotubes

**DOI:** 10.3390/molecules26247470

**Published:** 2021-12-10

**Authors:** Jun Wang, Yinfeng Chen, Peishi Yu

**Affiliations:** 1 School of Mechanical Technology, Wuxi Institute of Technology, Wuxi 214121, China; wangjun@wxit.edu.cn; 2 School of Mechanical Engineering, Jiangnan University, Wuxi 214122, China; yinfeng_chen@163.com

**Keywords:** vdW interaction, single-walled carbon nanotube, cohesive energy, vibration

## Abstract

Based on the van der Waals (vdW) interaction between carbon atoms, the interface cohesive energy between parallel single-walled carbon nanotubes was studied using continuous mechanics theory, and the influence of the diameter of carbon nanotubes and the distance between them on the cohesive energy was analyzed. The results show that the size has little effect on the cohesive energy between carbon nanotubes when the length of carbon nanotubes is over 10 nm. At the same time, we analyzed the cohesive energy between parallel carbon nanotubes with the molecular dynamics simulation method. The results of the two methods were compared and found to be very consistent. Based on the vdW interaction between parallel carbon nanotubes, the vibration characteristics of the two parallel carbon nanotube system were analyzed based on the continuous mechanical Euler-beam model. The effects of the vdW force between carbon nanotubes, the diameter and length of carbon nanotubes on the vibration frequency of carbon nanotubes was studied. The obtained results are helpful in improving the understanding of the vibration characteristics of carbon nanotubes and provide an important theoretical basis for their application.

## 1. Introduction

For small structures on the scale of nanometers, the intermolecular van der Waals (vdW) interaction can play a leading role in some cases [1]. Since their discovery, carbon nanotubes (CNTs) have shown great application prospects in various fields with their excellent physical and mechanical properties [2,3,4,5].

The molecular dynamics (MD) simulation software LAMMPS (Large-scale Atomic Molecular Massively Parallel Simulator) [6] has been widely used to predict the remarkable thermomechanical properties of CNTs, such as the influence of grain boundaries on the mechanical properties of polycrystalline carbon nanotubes [7] and the transversely isotropic thermal properties of carbon nanotubes containing vacancies [8,9]. The existing research results show that in a micro system composed of CNTs bundles, the van der Waals interaction between adjacent carbon tubes has a great impact on the mechanical behavior of the system [10,11,12,13,14,15].

With the influence of finite size and boundary effects, the internal vdW bonding energy has an important impact on the mechanical behavior of CNT bundle micro systems [16,17,18]. Therefore, clarifying the bond energy between tube bundles of finite size has important guiding significance for the design and application of microsystems [19,20,21,22,23,24,25].

In previous work, Zhao et al. obtained the analytical solution of the bonding energy per unit length of infinite parallel single-walled CNTs based on the continuous model, and analyzed the influence of CNT diameter on the bonding energy and the equilibrium distance of the interface [13]. However, due to the limited length of CNTs in actual microsystems, the scale effect of length on the cohesive energy between tubes needs to be further explored.

Zhang et al. [17] prepared suspended single-walled CNT array devices and found that this structure has unique mechanical behavior. Based on the molecular mechanics model, Chang [18] proposed an anisotropic shell model to reveal the mechanical properties of CNTs, and studied the young’s modulus, Poisson’s ratio and radial breathing mode of single-walled CNTs, which laid a foundation for further study of the influence of the bonding energy between parallel carbon tubes on microsystems. Rueckes et al. [26] studied a suspended single-walled CNT array and found that the array has good characteristics of switchable and bi-stable device elements, and the mechanical stability of this structure is determined by the vdW interaction between single-walled CNTs. Wang and Hu further studied the thermodynamic vibration between monolayer graphene sheets based on the nonlocal elastic plate model [27]. Parallel CNT systems have prospective application as micromechanical systems.

For nano-mechanical systems, the shear deformation effect has been studied recently in the literature. Al-Furjan et al. presented the vibrational characteristics of a rotating orthotropic piezoelectric nanodisk [28]. Li et al. analyzed and tested the quasi-static compression and hygrothermal stability of BMI/CE co-cured composite lattice cylindrical shell [29]. Zhang et al. designed and fabricated an ultra-lightweight beam string structure made of carbon fibre-reinforced polymer (CFRP) composites [30]. Dai et al. investigated the vibrations of non-polynomial viscoelastic composite open-type shells under residual stresses [31]. Zhang et al. dealt with the vibration and low-velocity impact responses of functionally graded graphene nanoplatelet-reinforced composite panels on a viscoelastic foundation [32].

Considering the vdW interaction energy between the CNTs, it is expected that the vibration characteristics of the system are significantly different from those of an independent single tube, but this has not yet been clarified. Therefore, in this paper, the corresponding theoretical analyses and numerical simulations are carried out for the above problems. The paper is divided into three parts. Firstly, the van der Waals interaction between carbon atoms is analyzed, secondly, the bonding energy between finite size single-walled CNTs is calculated, based on the continuous mechanics theory, and finally, the vibration characteristics of a parallel single-walled bi-CNT system are analyzed.

## 2. Quantification of the vdW Interaction between Carbon Atoms

The vdW interaction energy between the carbon atoms is expressed by Equation (1) [13,33],

(1) $$V(r)=4\varepsilon [{\left(\frac{\sigma }{r}\right)}^{12}-{\left(\frac{\sigma }{r}\right)}^{6}]$$

where *r* is the distance between atoms, *ε* is the potential well depth with the value of 2.8437 MeV and *σ* is a reference value determined as 3.4 Å the equivalent distance between the carbon atoms.

**Figure 1:**
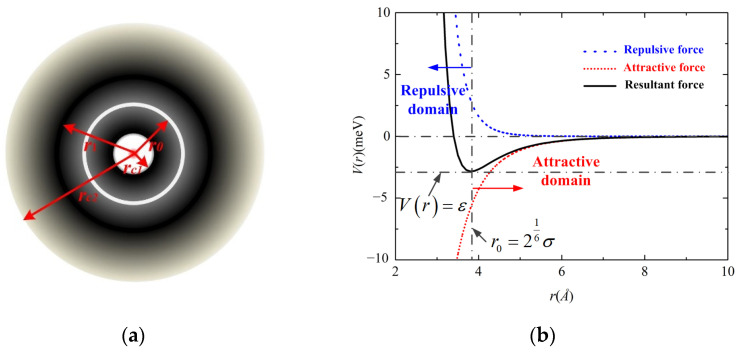
The vdW interaction between atoms: (**a**) the distribution diagram energy density surrounding the atom; (**b**) the interaction variation with the distance between two atoms.

Based on Equation (1), the vdW energy variation with the distance between two carbon atoms is shown in Figure 1. For *r* < *r*_0_, the repulsive force is dominant, defining the repulsive domain, whereas the attractive force is dominant for *r* > *r*_0_, forming the attractive domain. The blue dash, red dot and black solid lines in Figure 1b represent the repulsive, attractive and resultant forces between the two interacting atoms, respectively. The lowest point of the resultant force is the equilibrium position, which is determined by the energy-minimization through $\frac{\partial^{2}V(r)}{\partial r^{2}}=0$, leading to *r*_0_ = 3.8165 Å.

## 3. Cohesive Energy between Two Finite-Length Parallel CNTs

**Figure 2:**
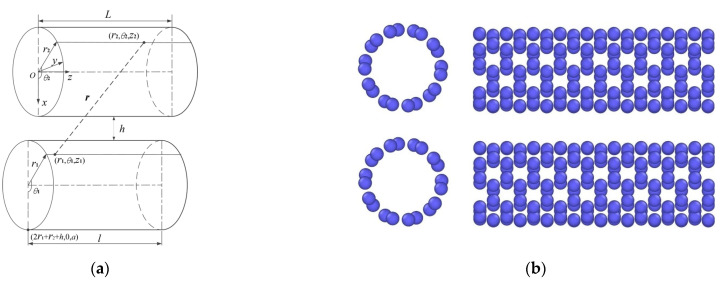
The (**a**) continuum model and coordinate system of two parallel single-walled CNTs and the (**b**) atom models of the MD simulations.

The continuum model and coordinate system of two parallel single-walled CNTs are established as shown in Figure 2a. The radii of the CNTs are *r*_1_ and *r*_2_ respectively, and the shortest distance between parallel CNTs is *h*.

Molecular dynamics (MD) simulations were conducted using the LAMMPS software package [6] to verify the analytical model, as shown in Figure 2b. The adaptive intermolecular reactive empirical bond order (AIREBO) potential was used [34], which evaluates the covalent carbon–carbon bonding interaction by the well-established REBO potential, and the non-bonded van de Waals interaction was described by the 12-6 Lennard-Jones (LJ) potential (σ = 0.34 nm and ε = 0.0028 eV) [35,36]. ReaxFF was mainly used for the chemical reaction atomic simulations, as the Tersoff potential cannot describe the non-bonded van de Waals interactions between CNTs. The AIREBO potential function with an LJ cut-off radius of 10.2 Å was adopted in all the simulations [37,38]. The Polak–Ribiere version of the conjugated gradient algorithm [39] was used to optimize the initial positions of atoms. The temperature of system was controlled using Nose–Hoover thermal bath coupling [40,41] (coupling constant 0.1 ps, time step 0.5 fs) for 0.5 ns. The time step used in the simulations was set at 0.5 fs.

For the two parallel CNTs with finite length, the distribution along the horizontal direction is nonuniform, and can be determined by integration of the following Equation (2),

(2) $$\begin{array}{l} \phi_{total}=\int_{A_{1}}\rho_{c}V(r)dA_{1}\int_{A_{2}}\rho_{c}dA_{2}\\ =\rho_{c}^{2}\int_{0}^{2\pi }r_{1}V(r)d\theta_{1}\int_{a}^{a+l}dz_{1}\int_{0}^{2\pi }r_{2}d\theta_{2}\int_{0}^{L}dz_{2} \end{array}$$

where,

$$\begin{array}{l} r^{2}={\left(r_{1}\cos\theta_{1}+r_{1}+r_{2}+h-r_{2}\cos\theta_{2}\right)}^{2}+\\ {\left(r_{1}\sin\theta_{1}-r_{2}\sin\theta_{2}\right)}^{2}+{\left(z_{1}-z_{2}\right)}^{2} \end{array}$$

and *ρ*_c_ is the area density of the atom number in the wall (number of atoms per unit area in the wall), *A*_1_ and *A*_2_ are the out-wall areas for the two parallel CNTs. Substituting the Equation (1) to Equation (2), leads to

(3) $$\phi_{total}=4\varepsilon \sigma^{6}\rho_{c}^{2}r_{1}r_{2}[\sigma^{6}B_{1}-B_{2}]$$

where *B*_1_ is determined by,

(4) $$\begin{array}{l} B_{1}=\int_{0}^{2\pi }\frac{d\theta_{1}\int_{a}^{a+l}dz_{1}\int_{0}^{2\pi }d\theta_{2}\int_{0}^{L}dz_{2}}{{\left(\begin{array}{l} {\left(r_{1}\cos\theta_{1}+r_{1}+r_{2}+h-r_{2}\cos\theta_{2}\right)}^{2}\\ +{\left(r_{1}\sin\theta_{1}-r_{2}\sin\theta_{2}\right)}^{2}+{\left(z_{1}-z_{2}\right)}^{2} \end{array}\right)}^{6}}\\ =\frac{\pi^{2}\cdot l\cdot L}{4}\sum_{i=1}^{n}\sum_{j=1}^{n}\sum_{k=1}^{n}\sum_{p=1}^{n}w(i)w(j)w(k)w(p)H_{1}(t) \end{array}$$

in which,

(5) $$H_{1}(t)=\frac{1}{{\left(\begin{array}{l} (r_{1}\cos(\pi +\pi t(i))+r_{1}+r_{2}+h\\ -r_{2}\cos(\pi +\pi t(k)){)}^{2}\\ +(r_{1}\sin(\pi +\pi t(i)\\ -r_{2}\sin(\pi +\pi t(k)){)}^{2}\\ +{\left(a+\frac{l}{2}+\frac{l}{2}t(j)-\frac{L}{2}-\frac{L}{2}t(p)\right)}^{2} \end{array}\right)}^{6}}$$

Similarly, *B*_2_ is calculated by,

(6) $$\begin{array}{l} B_{2}=\int_{0}^{2\pi }\frac{d\theta_{1}\int_{a}^{a+l}dz_{1}\int_{0}^{2\pi }d\theta_{2}\int_{0}^{L}dz_{2}}{{\left[{\left(r_{1}\cos\theta_{1}+r_{1}+r_{2}+h-r_{2}\cos\theta_{2}\right)}^{2}+{\left(r_{1}\sin\theta_{1}-r_{2}\sin\theta_{2}\right)}^{2}+{\left(z_{1}-z_{2}\right)}^{2}\right]}^{3}}\\ =\frac{\pi^{2}\cdot l\cdot L}{4}\sum_{i=1}^{n}\sum_{j=1}^{n}\sum_{k=1}^{n}\sum_{p=1}^{n}w(i)w(j)w(k)w(p)H_{2}(t) \end{array}$$

where,

(7) $$H_{2}(t)=\frac{1}{{\left(\begin{array}{l} (r_{1}\cos(\pi +\pi t(i))+r_{1}+r_{2}+h\\ -r_{2}\cos(\pi +\pi t(k)){)}^{2}\\ +(r_{1}\sin(\pi +\pi t(i)\\ -r_{2}\sin(\pi +\pi t(k)){)}^{2}\\ +{\left(a+\frac{l}{2}+\frac{l}{2}t(j)-\frac{L}{2}-\frac{L}{2}t(p)\right)}^{2} \end{array}\right)}^{3}}$$

in which, *t* is the value corresponding to the given Gaussian point, *n* is the number of Gauss points, and *W* is the Gauss coefficient corresponding to the Gauss point.

For *a* = 0 and *l* = *L*, the average cohesive energy per unit length from the vdW interactions is determined by,

(8) $$\phi_{ave}=\frac{\phi_{total}}{L}=\frac{4\varepsilon \sigma^{6}\rho_{c}^{2}r_{1}r_{2}[\sigma^{6}A_{1}-A_{2}]}{L}$$

**Figure 3:**
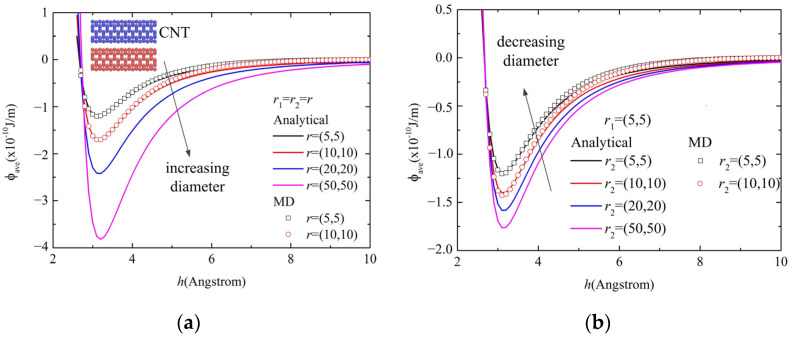
Size effect of cohesive energy of parallel CNTs with (**a**) the same diameter and (**b**) different diameters.

The size-dependence of the cohesive energy between the two parallel CNTs is shown in Figure 3. It was found that the results obtained by the proposed analytical model for a pair of parallel CNTs with the length of 50 Å show high agreement with those of MD simulations based on the models shown in Figure 2b. Additionally, the influence of the interval distance on the cohesive energy shows a non-monotonic tendency, which is quantified as shown in Figure 3. Here *h* is the inter-wall distance between two parallel CNTs. The position changes with different CNT diameters. The results in Figure 3 suggest that the energy-optimized distance *h* is independent of CNT diameters.

## 4. The Vibration Characteristic of the Two Parallel CNTs

**Figure 4:**
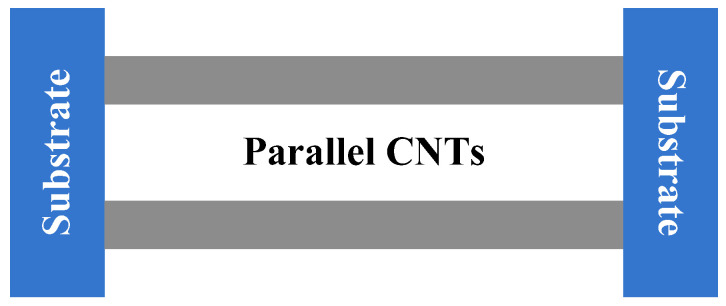
Continuum model for vibration analysis of parallel CNTs with constrained substrates.

The ends of the two parallel CNTs are fixed on substrates, as shown in Figure 4. The vibration characteristic of the two parallel CNTs is analyzed based on the continuum Euler beam model. Accordingly, the vibration equations of the system were obtained following [42],

(9) $$\begin{array}{l} E_{1}I_{1}\frac{\partial^{4}w_{n1}}{\partial x^{4}}+\rho_{1}S_{1}\frac{\partial^{2}w_{n1}}{\partial t^{2}}=K_{1}(w_{n1}-w_{n2})\\ E_{2}I_{2}\frac{\partial^{4}w_{n2}}{\partial x^{4}}+\rho_{2}S_{2}\frac{\partial^{2}w_{n2}}{\partial t^{2}}=-K_{1}(w_{n1}-w_{n2}) \end{array}$$

where *E_i_* is the Young’s modulus for the *i*th CNT, here we use the well accepted value as *E*_1_ = *E*_2_ = 1 TPa, *I_j_* is the moment of the inertia of the *j*th CNT and is defined as *I*_1_ = *I*_2_ = 5.21 × 10^−38^ m^4^, *ρ_k_* is the mass density of the *k*th CNT and set as *ρ*_k_ = *ρ*_k_ = 1.3 g/cm^3^, *S_q_* is the cross-section area of the *q*th CNT and *K*_1_ is the vdW interaction coefficient between parallel CNTs, determined by $K_{1}=\frac{\partial^{2}\phi_{ave}}{\partial h^{2}}$.

To solve the above equation, the vibration mode functions were assumed to be,

(10) $$\begin{array}{l} w_{n1}=A_{n1}\sin(\frac{n\pi x}{L})e^{j\omega_{1}t}\\ w_{n2}=A_{n2}\sin(\frac{n\pi x}{L})e^{j\omega_{2}t} \end{array}$$

where *A_n_*_1_ and *A_n_*_2_ are the vibration amplitudes for the two CNTs.

Substituting the Equation (10) to (9), we have,

(11) $$\begin{array}{l} \omega_{n1}^{2}=\frac{1}{2}(\alpha_{n}-\sqrt{\alpha_{n}^{2}-4\beta_{n}})\\ \omega_{n2}^{2}=\frac{1}{2}(\alpha_{n}+\sqrt{\alpha_{n}^{2}-4\beta_{n}}) \end{array}$$

where,

(12) $$\begin{array}{l} \alpha_{n}=\frac{E_{1}I_{1}\lambda_{n}^{4}+K_{1}}{\rho_{1}S_{1}}+\frac{E_{2}I_{2}\lambda_{n}^{4}+K_{1}}{\rho_{2}S_{2}}\\ \beta_{n}=\frac{E_{1}I_{1}E_{2}I_{2}\lambda_{n}^{8}}{\rho_{1}^{2}S_{1}S_{2}}+K_{1}\lambda_{n}^{4}\frac{E_{1}I_{1}+E_{2}I_{2}}{\rho^{2}S_{1}S_{2}} \end{array}$$

in which *λ_n_* is determined by,

(13) $$\frac{d^{4}Y(x)}{dx^{4}}=\lambda_{n}^{4}Y(x),Y(x)=\sin(\frac{n\pi x}{L})$$

leading to *λ_n_* = *n*π/*L*.

**Figure 5:**
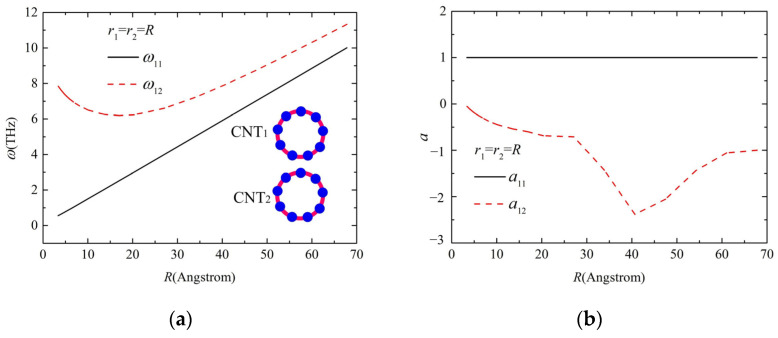
Vibration of the two parallel CNTs with the same diameter: (**a**) the resonance frequency and (**b**) amplitude ratio of CNTs.

Figure 5 shows the resonant frequency and the amplitude ratio of the two parallel CNTs with the same diameter. The resonant frequency in Figure 5a increases nonlinearly with the tube diameter and reaches a minimum for a radius of 15 Å. The amplitude ratio reaches the lowest point for a radius of 40 Å, as shown in Figure 5b.

## 5. Discussion

**Figure 6:**
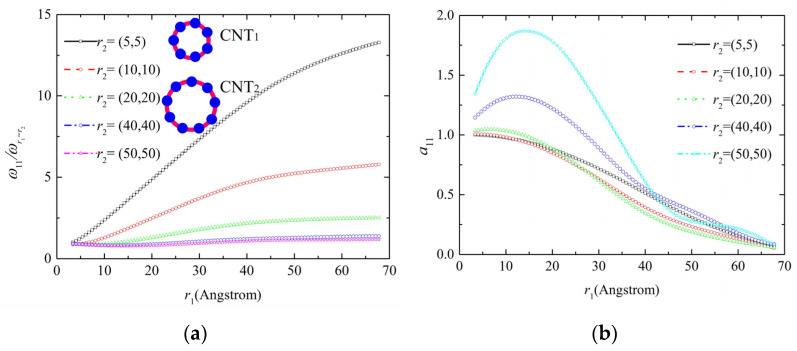

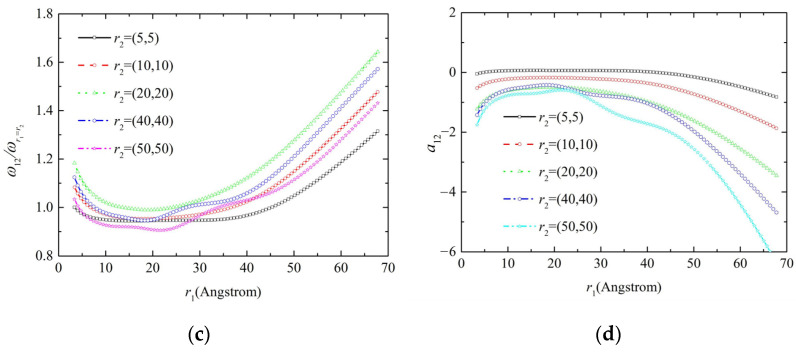
Vibration characteristic of the two parallel CNTs with different diameters: (**a**) resonance frequency under low frequency, (**b**) amplitude ratio of CNTs under low frequency, (**c**) resonance frequency under high frequency, (**d**) amplitude ratio of CNTs under high frequency.

For two parallel CNTs with different tube diameters, the vibration frequencies are predicted by the analytical model as shown in Figure 6. The resonance frequency in Figure 6a for low-frequency vibration decreases with the increase in tube diameter. For high-frequency vibration in Figure 6c the resonance frequency reaches the maximum value when r_2_ = (20,20), which is among the several prediction results including r_2_ = (5,5)~(50,50). The amplitude ratio in Figure 6b,d show remarkable dependence and significant nonlinearity with the change in the diameters of the two CNTs.

**Figure 7:**
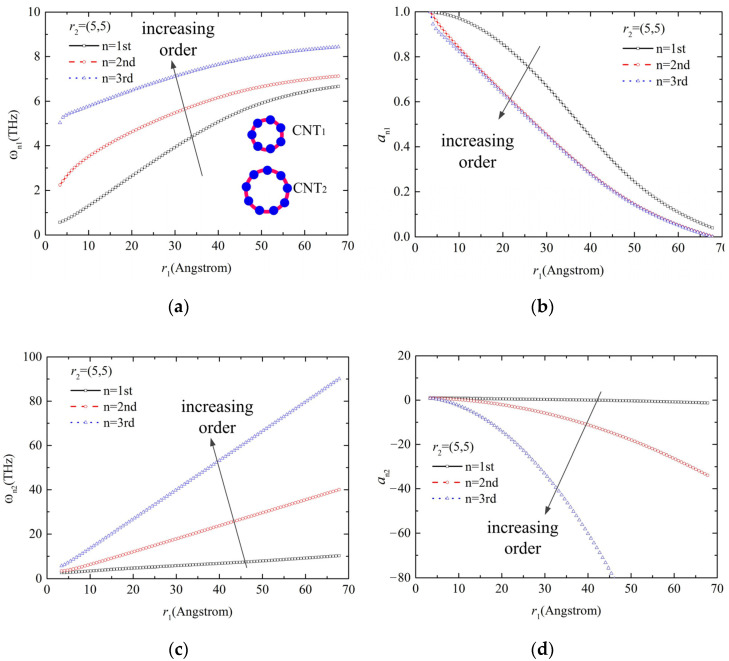
Effect of order on the vibration of CNTs with different diameters: (**a**) the increasing tendency for the resonant frequency with the increase in order under low frequency, (**b**) the decreasing dependence of amplitude ratio on the order under low frequency, (**c**) the increasing tendency for the resonant frequency with the increase in order under high frequency, (**d**) the decreasing dependence of amplitude ratio on the order under high frequency.

The influence of vibration modes (takes the first three orders into account) on the resonant frequency for the bi-CNT system with different tube diameters was obtained and is shown in Figure 7. It can be seen from Figure 7a,c that the order has a significant influence on the resonant frequency of carbon nanotubes, which show an increasing tendency in the resonant frequency with the increase in order. The amplitude ratio presents a decreasing dependence on the order, as shown in Figure 7b,d. This confirms that the system is nonlinear because the vdW interaction is nonlinear.

The proposed model focuses on the vibration of CNTs with large slenderness ratios. Thus, the deformation of the cross section is neglected in the continuum beam model. However, if the prerequisite of the large slenderness is not satisfied (for short CNT with large radius), the geometric nonlinearity would be influential on the vibration properties and cannot be neglected. The scope of applications of the continuum mechanics model at the nano scale is always an important topic and has been the subject of many studies in the field [11,13,42]. Here we proposed a beam model with the vdW interaction to analyze the dynamic behavior for a pair of parallel CNTs, and the high agreement of the results between the proposed beam model and the MD simulations indicates the high applicability of the continuum model. Therefore, we suggest that overall vibration modes for nano tubes (with a large ratio of length/radius > 5) can be effectively analyzed by the continuum model for the dominant interaction (vdW, for instance). Since the CNTs are a promising material for constructing nano electromechanical systems, the proposed model provides an analytical model for designing the performance of CNT-based devices such as sensors, actuators, intelligent machines and so on.

## 6. Conclusions

Based on the continuum mechanics model and the Gauss integral method, an analytical solution of the cohesive energy between parallel single-walled CNTs was obtained. On this basis, the vibration characteristics of a bi-CNT system were studied. Compared to the MD simulations, the continuum model is capable of providing more comprehensive results with a much lower computation cost. The main findings are concluded as follows:

(1) The analytical solutions for the vdW interaction between CNTs were obtained, which show remarkable nonlinear dependence on the interval distance between the tubes;
(2) The cohesive energy between adjacent CNTs varies nonlinearly with increasing tube diameter;
(3) The vibration frequency of the bi-CNT system is remarkably affected by the vibration mode and the tube diameter, which show different dependence on low-frequency and high-frequency vibrations.

## Data Availability

Not applicable.

## Sample Availability

Samples of the compounds are not available from the authors.
